# Report of Am. Ins. of Homœopathy

**Published:** 1881-01

**Authors:** Ad. Lippe


"TRANSACTIONS OF THE AMERICAN INSTITUTE OF HOMŒOPATHY, SESSION 1880."—The members of the American Institute of Homœopathy will surely express their thanks to their secretary, Dr. T. C. Burgher of Pittsburg, for his prompt publication of the Transactions of 1880. Early in September every member of the Institute received his copy. Dr. Burgher, unmindful of disgraceful precedents, gives us a more truthful report of the transactions of the Institute at a very much earlier date than we were accustomed to receive at a late date. The deviations from a perfectly correct report of the Transactions as discovered by Dr. Pearson, (Washington) and published in the Nov. No. of the *Advance* can hardly be charged to Dr. Burgher, who, contrary to precedents, reported all the transactions without assuming, as his predecessor did, the right to trim the transactions to please designing men. No doubt Dr. Burgher will have an eye on the transcribed transactions and in future not only detect intermeddlers and wilful perverters of the truth, but if detected, expose them.

The reports of the transactions of the Institute in 1876 and 1879 are still due! The late secretary, failing to keep his solemn promises, recommended his successor, to whom he professed to have given all the MS. in his possession; his successor was endorsed on account of the aptitude he had shown when he brought out the "Transactions of the State Society of Pennsylvania;" he had shown his "aptitude" by aiding to "spirit away" a paper which Dr. Ad. Lippe had presented to the State Society with the provings of Lac can. the results of long and protracted study, showing its relation to other remedies. We hope the Institute will fully appreciate Dr. Burgher's promptitude and efforts to break through baneful precedents, that is, the right the former secretary and his near friends wrongfully assumed to serve and act as "Censors," scissors in hand to trim papers, or if it were not considered prudent, for private reasons or as an auxiliary and supplementary measure to extinguish true Homœopathy, to spirit them away, in the waste-basket or fire-place. Let Dr. Burgher be honored by a re-election to the post he so well fills, just as long he will be good enough to serve us.

"The Transactions of the Homœopathic Medical Society of Pennsylvania," are in the possession of the members of that society in less than eighty days after it was held. A general index of the "Transactions," for the last fourteen years is also added. A determined effort to publish the transactions truthfully, in full, and at an early date will undoubtedly make members more willing to attend the annual meetings and furnish carefully written, instructive papers. The thanks of the Society are due Drs. Z. T. Miller, R. E. Caruthers, I. F. Cooper, T. M. Strong, the Committee of Publication.—AD. LIPPE.

